# Tracking spasticity dynamics in hemiparetic stroke survivors following cyproheptadine administration: a pilot study using controlled varying tendon indentation depths

**DOI:** 10.3389/fstro.2025.1534600

**Published:** 2025-04-23

**Authors:** Sungjin Bae, Matthieu K. Chardon, Elliot J. Roth, William Z. Rymer, Nina L. Suresh

**Affiliations:** ^1^Department of Physical Medicine and Rehabilitation, The Feinberg School of Medicine, Northwestern University, Chicago, IL, United States; ^2^Shirley Ryan AbilityLab, Chicago, IL, United States; ^3^Department of Neuroscience, Feinberg School of Medicine, Northwestern University, Chicago, IL, United States

**Keywords:** stretch reflex threshold, deep tendon reflex, stroke rehabilitation, spasticity, cyproheptadine HCl

## Abstract

This study evaluates the potential of the Linmot^®^ tapper as a precise tool for tracking spasticity changes in hemiparetic stroke survivors following cyproheptadine HCl administration. Spasticity, a significant health concern among stroke survivors, is characterized by increased muscle tone due to upper motor neuron dysfunction. Conventional clinical assessments, such as the Modified Ashworth Scale (MAS), often lack the sensitivity to accurately monitor treatment. In this study, we utilized the Linmot^®^ tapper to assess the stretch reflex threshold (SRT) in three stroke survivors and one control subject by progressively altering tendon indentation to change muscle length. The SRT was defined as the indentation depth at which consistent reflex responses of the biceps brachii were observed, as indicated by reflex force or rectified integrated EMG (RIEMG) signals. Measurements were taken at baseline and at 2, 4, and 6 h after drug administration. Results showed significant increases in SRT following cyproheptadine administration, indicating reduced motor neuron excitability and highlighting the drug's effect on spasticity. Both reflex force and RIEMG data consistently captured these changes, while MAS grades remained unchanged. The high correlation between SRTs derived from force and EMG further supports the tool's accuracy in detecting subtle neuromuscular changes. These findings highlight that the Linmot^®^ tapper offers a precise, quantitative method for monitoring spasticity dynamics, providing a more accurate alternative to conventional clinical assessments and demonstrating potential for enhancing stroke rehabilitation strategies.

## 1 Introduction

Spasticity, a significant health issue for chronic stroke survivors, is defined as a disorder of sensorimotor control resulting from an upper motor neuron lesion (Pandyan et al., 2005). It is characterized by increased involuntary velocity-dependent tonic stretch reflexes with exaggerated tendon jerks resulting from hyper-excitability of the stretch reflex (Lance, 1980). Spasticity-induced physical limitations, such as diminished joint mobility, loss of fine motor skills, and abnormal limb positioning, can progressively hinder functional abilities including mobility, transfers, and activities of daily living. Effective management of spasticity is crucial for preventing complications, enhancing clinical outcomes, and improving motor function in chronic stroke survivors. This requires proper evaluation and accurate assessment of spasticity, especially within the context of interventional treatments.

Reliable and high-resolution evaluation of spasticity before and after an interventional treatment is essential for determining the effective timing and dose for individual stroke survivors and tracking the intervention's progress. Conventional clinical spasticity assessments such as the Modified Ashworth Score (MAS) and the Tardieu scale, which have both been widely used for over 30 years, are qualitative and thereby subjective and have been reported to vary significantly between different raters and even within the same rater at other times (Alibiglou et al., 2008; Haugh et al., 2006). Recognizing the critical role of spasticity evaluation in rehabilitation, numerous efforts have been undertaken to devise efficient and reliable technological methodologies to develop quantitative spasticity assessments (Sin et al., 2019; Condliffe et al., 2005; Starsky et al., 2005; Calota et al., 2008; Choi et al., 2018; Centen et al., 2017; Dehem et al., 2017; Park et al., 2019).

Several studies have utilized the stretch reflex threshold (SRT) to correlate spasticity for reliable and precise measurement of the spasticity (Calota et al., 2008; Chardon et al., 2014; Lemoyne et al., 2011). The SRT represents the minimum muscle length or joint angle at which a muscular reflex response is recorded to stretch (Feldman, 2015; Raptis et al., 2010). In our previous study, we used a tendon tapper with a high-precision linear actuator to accurately evaluate the SRT in the biceps brachii of chronic stroke survivors through progressive indentation of the biceps tendon (Li et al., 2021). The indentation depth correlates with changes in muscle length (Chardon et al., 2020). The results from our previous study showed that the SRT was lower on the affected side of stroke survivors with spasticity as compared to the contralateral side (Chardon et al., 2014).

The difference in SRT responses between sides of stroke survivors may be linked to hyperactivity in spinal motoneurons. This increased activity is likely due to a loss of control of supraspinal centers. However, the exact processes behind muscle tone and spasticity after upper motor neuron injury must be better understood (Li et al., 2021). A potential mechanism is the deregulation of monoaminergic neuromodulators, such as serotonin, which significantly modulate spinal motoneuron excitability. *In vitro*, animal studies have shown that administering these neuromodulators raises motoneuron resting potential, increasing firing activity and ultimately generating exaggerated and sustained reflex responses characteristic of spasticity (Hornby et al., 2002; Wang and Dun, 1990). Therefore, inhibiting the activity of these neuromodulators by administering pharmacological agents may help reduce involuntary muscle activation.

One such agent is cyproheptadine hydrochloride (HCl), a serotonergic antagonist. It has been shown in human subjects to reduce clonus and muscle spasms in patients with spinal cord injuries (SCI) while producing a mild decrease in dynamic muscle strength (Wainberg et al., 1990; Nance, 1994). In stroke survivors, the use of cyproheptadine HCl was associated with a reduction in hypertonicity or spasticity, as evidenced by a decline in muscle relaxation time, and this effect was achieved without affecting voluntary strength (Kamper et al., 2022). It should, therefore, be expected that the SRT will change after a stroke subject is administered cyproheptadine HCl.

The objective of this study was to examine how the precision tendon tapper, we will designate as the Linmot^®^ tapper, can measure the SRT, a correlate of spasticity, and precisely track changes before and within 6 h after cyproheptadine HCl administration in chronic stroke survivors. A neurologically intact control was added to measure the Linmot^®^ tapper's baseline variability across repeated testing sessions and to isolate the effects of the drug in the presence of spasticity. The Linmot^®^ tapper can change muscle length by systematically adjusting the tendon indentation depth in sub-millimeter increments upon which superimposed brief controlled taps at each depth attempt to elicit a reflex response (Chardon et al., 2015). This allows for assessing reflex excitability changes at different time points following the administration of a serotonin antagonist. We hypothesize that the Linmot^®^ tapper can accurately track changes in the SRT following cyproheptadine HCl administration and that the SRT will increase upon administration. In this manner, we can precisely track spasticity management with interventional treatments.

## 2 Methods

### 2.1 Participants

In this study, we recruited three hemi-spastic stroke survivors (aged 60–68 years, 9–15 years post-stroke) and one age-matched neurologically intact control i.e., with no diagnosed neurological disorder or no clinical diagnosed spasticity (MAS of 0). They were recruited from the Clinical Neuroscience Research Registry at the Rehabilitation Institute of Chicago. The stroke survivors were tested on their stroke-affected side, while the control subject was tested on their dominant side. The stroke survivors were tested on their affected side (contra-lesional side) while the control subject was tested on their dominant side. Stroke survivors met the inclusion criteria of being at least 6 months post-stroke, having sustained a single hemispheric stroke, not participating concurrently in other upper-extremity research studies, and having a MAS score of ≥1. Patients with a history of prior neurological disorders or those receiving other pharmacological treatments for spasticity or botulinum toxin were excluded. The Northwestern University Institutional Review Board approved the study, and all subjects provided informed consent.

### 2.2 Experimental setup

#### 2.2.1 Linmot^®^ tapper

**Figure 1:**
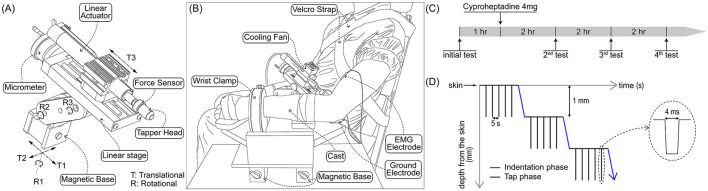
Schematics representation of the experimental device and protocol. **(A)** An isometric view of the device and its degrees of freedom. The device comprises a force sensor, a linear actuator, and custom frames equipped with a micrometer and a magnetic base. **(B)** Experimental setup. The forearm is stabilized using a fiberglass cast and wrist clamp. The tip of the tapper head is precisely positioned over the distal tendon of the biceps brachii, with sub-millimeter precision maintained via manual operation of the micrometer. **(C)** The schematic representation of the experimental protocol. The initial testing is conducted 1 h prior to the administration of a single 4mg dose of cyproheptadine HCl, followed by three tests at 2-h intervals. **(D)** Indentation & tapping sequence. Starting at the skin surface, the protocol involves a series of five taps (black line) alternating with 1mm indentations (blue line), progressing until the device tip reaches a depth of 20mm. Both taps and indentations are spaced at 5-s intervals. Each individual tap (dotted line), designed to be 4 ms in duration and 1mm in depth, has a rectangular shape to minimize its impact on the reflex response, which typically occurs within 15–20 ms.

As detailed in our initial study, the Linmot^®^ tapper evaluates spasticity by measuring the muscle stretch reflex threshold through controlled indentation and taps to the distal biceps tendon (Chardon et al., 2014). The device features a high-resolution linear actuator (Linmot S.A., Spreitenbach, Switzerland) and a custom frame with six degrees of freedom (three rotational and three linear) for precise spatial positioning (Figure 1A). One linear axis, aligned with the actuator, is manually controlled using a micrometer (Velmex, Bloomfield, NY, USA), achieving sub-millimeter accuracy (up to 1/10 mm) to enable progressive 1 mm indentations for precise adjustments of muscle length. The motor controller integrates a PID system to ensure accurate, repeatable movements. A single-axis force sensor (Sensotec Model 31, Honeywell, Columbus, OH, USA), capable of measuring forces up to 50 N in both compression and tension, is positioned directly behind the tapper head to measure reaction forces during the experiment simultaneously.

#### 2.2.2 Experimental protocol

To track the change in SRT following the administration of the cyproheptadine HCl, we conducted four tests: the initial 1-h test before drug administration, followed by three subsequent tests conducted at 2-h intervals post-administration (Figure 1C). A study physician administered a single 4 mg dose of the cyproheptadine HCl, previously shown to be effective for treating spasticity in stroke survivors (Kamper et al., 2022).

Subjects were seated in a Biodex chair (Shirley, NY, USA) and secured with Velcro straps across the torso. The lower arm was immobilized using a fiberglass cast, which was clamped to a custom magnetic base secured to a steel table before it cured (Figure 1B). Using a manual goniometer, we adjusted the limb to the following positions: shoulder abduction at 45°, shoulder flexion at 20°, elbow extension at 120°, and lower arm abduction at 45° (Chardon et al., 2014). After the initial test, the cast was cut, removed, and marked for repositioning. The same cast was reapplied to the remaining three tests to ensure the arm was consistently positioned and postured.

The Linmot^®^ tapper was oriented at 90-degree angle to the biceps tendon, with the depth zeroed at the skin's surface. The position was continuously monitored via force sensor measurements to ensure consistent stimuli and reflex measurements. Medical tape on the apparatus was used to consistently position the Linmot^®^ tapper at the exact location for each test. The position and orientation of the Linmot^®^ tapper were fixed for the initial test and remained unchanged for the subsequent three tests to confirm repeatability. Blood pressure and heart rate were measured just before every test to ensure the participant's health and safety following drug administration. Additionally, a physical therapist measured the MAS grade before each test to assess spasticity.

#### 2.2.3 Data acquisition

During the experiment, two signals were recorded: The contact/reaction force on the biceps tendon using the force sensor at the device's tip and the EMG signals captured by surface bipolar electrodes placed on the lateral head of the biceps brachii. A Delsys Bagnoli system (Boston, MA, USA) was used to acquire EMG signals, while Spike 2 software (CED, Cambridge, UK) was used to collect and synchronize all data with a sampling frequency of 2,000 Hz.

With the subject position secured and the EMG electrodes and device in place, we began the preload and tapping protocol with the tip of the linear actuator. As detailed in our previous work (Chardon et al., 2014), a unique tapping procedure with two phases, indentation and tapping, was employed (Figure 1D). Briefly, the indentation phase changed the position of the tip of the device started from the skin surface. A 1 mm indentation was manually conducted to prevent slippage from the tendon. After reaching every new depth from the surface (0 mm to 20 mm), the tap phase was initiated, consisting of five taps with an interval of 5 s. Each tap, with a 1 mm depth and a 4 ms trapezoidal pulse, was designed to avoid interfering with the reflex within 15–20 ms. These two phases were repeated until the participants tolerated a total indentation distance of at least 20 mm. The subject was instructed to relax/not intervene and remain passive throughout the experiment with continuous monitoring of the force and EMG signals to ensure the same. Before the start of each tendon tap, baseline EMG checks were performed.

### 2.3 Data analysis

#### 2.3.1 Signal processing

**Figure 2:**
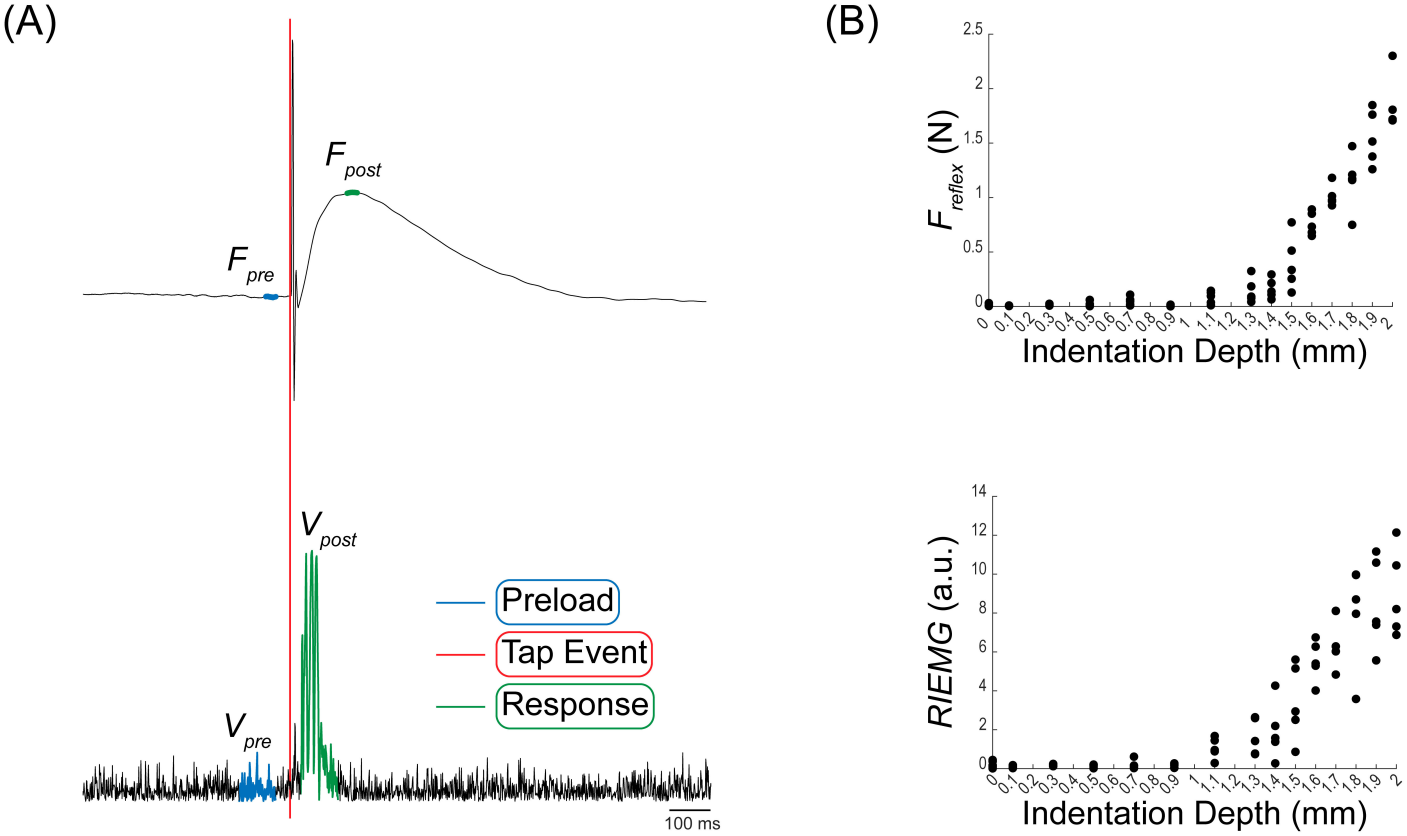
Example of the force and EMG measures. **(A)** Raw force traces from the force sensor and rectified biceps EMG during a single reflex response. Both signals segmented into two phases: the Preload phase (blue) before the tap event (red), and the Response phase (green) after the tap event indicating the reflex response. **(B)** Reflex force and rectified integrated EMG for each indentation depth. The reflex force (F_reflex_) is derived from the average preload force (*F*_pre_) and average of maximum response force (*F*_post_). Rectified integrated EMG (RIEMG) is calculated from rectified integrated preload EMG (*V*_pre_) and the rectified integrated response EMG (*V*_post_).

The reflex force (*F*_reflex_) and rectified integrated EMG (RIEMG) were analyzed to identify and track changes in the stretch reflex response. A typical example of measured data is shown in Figure 2A, which displays the raw force traces from the force sensor (top) and rectified biceps EMG (bottom) during a reflex response at a 16 mm indentation depth. A low-pass Butterworth filter with a 600 Hz cutoff frequency was applied to the force data to remove high-frequency noise. The EMG was filtered using a Butterworth bandpass filter spanning 20–450 Hz, then amplified with a gain of 100. The signals were divided into two phases: the preload phase, occurring before the tap event, and the response phase, following the tap event (Figure 2A). The preload phase reflects the tendon's resting state, while the response phase captures the reflex response.

#### 2.3.2 Reflex force and RIEMG calculation

To calculate the *F*_reflex_ and the RIEMG, we extracted preload and response phase value from the force (*F*_pre_ and *F*_post_) and the EMG (*V*_pre_ and *V*_post_) value. First, *F*_reflex_ was calculated by subtracting the preload force (*F*_pre_) from the response force (*F*_post_), thereby assessing the relative force difference between these two phases. *F*_pre_ is determined as the mean value over a 10 ms window preceding the tap (blue line in Figure 2A). Similarly, *F*_post_ is computed as the mean force over on 10 ms window centered on the peak response value (green line in Figure 2A). Next, RIEMG was calculated using the equation:


(1) $$RIEMG=\frac{\sum V_{post}-\sum V_{pre}}{\sum V_{pre}}$$

*V*_pre_ and *V*_post_ represent the rectified voltage values during the preload and response phase, respectively. RIEMG offers a normalized measure of muscle activity change by expressing post-tap EMG activity relative to pre-tap activity. The integration window length for this calculation varied from 50 to 90 ms, selected based on the onset and duration variations of EMG response among participants. It is important to note that while the window length varied among participants, the pre- and post-tap windows were kept the same length for each participant. The onset and duration of the EMG response were based on a statistical estimation of baseline EMG noise, using a threshold of the mean plus three standard deviations (mean + 3 SD). Since our protocol involved five taps at each indentation depth, five sets of *F*_reflex_ and RIEMG are obtained for each depth accordingly (Figure 2B).

#### 2.3.3 Stretch reflex threshold estimation

Using the extracted *F*_reflex_ and RIEMG values, we calculated the ***SRT***. The SRT in this study is defined as the indentation depth where reflex responses of the biceps brachii to taps are consistently recorded, as evidenced by *F*_reflex_ or RIEMG. We employed a non-parametric bootstrapping to estimate SRT using a piecewise linear regression (Ashworth, 1964). To create robust datasets, four values were randomly selected from each set of five taps at every indentation depth, creating an *m*-by-*n* matrix (where *m* is the chosen value and *n* represents the indentation depths). This random selection was repeated for 100,000 iterations, resulting in 100,000 such matrices. We applied piecewise linear regression to each of the 100,000 sets to identify the SRT, which was determined as the change point where the slope of the regression line shifted. We implemented a custom linear regression function “fun” in MATLAB (Equation 2) to capture the relationship of the *F*_reflex_ or REIMG (dependent variable) with the indentation depth (independent variable), allowing for a single potential change point. This function estimates the intercept representing the initial value, the change point depth where the slope potentially changes, and the slopes before and after the change point.


(2) $$f(x)=\;\{\begin{array}{cc} \theta_{1}+\theta_{3}x, & \text{if }x\leq \theta_{2}\\ \theta_{1}+\theta_{3}\theta_{2}+\theta_{4}\left(x-\theta_{2}\right), & \text{if }x>\theta_{2} \end{array}$$

Where θ_1_ is the intercept, θ_2_ is the change point, θ_3_ is the slope before the change point, and θ_4_ is the slope after the change point. The least squares curve fitting approach was used to optimize the model fit to the data, providing initial parameter estimates and setting realistic boundaries for the parameters.

#### 2.3.4 Statistical analysis

Using a custom MATLAB script, we calculated the mean and the 2.5th and 97.5th percentiles of the 100,000 change points to determine the 95% confidence interval. To assess significant differences among time points (pre-medication, 2, 4, and 6 h post-medication), we compared the confidence intervals for the change points at each time point.

Statistical significance was determined by assessing whether the confidence intervals overlapped, with non-overlapping intervals indicating significant differences between time points (Young and Lewis, 1997; Mooney et al., 1993). This approach suggests that when the confidence intervals do not overlap, the actual values of the change points likely differ due to the medication's effect over time. By interpreting non-overlapping confidence intervals as evidence of significant differences, we infer that the observed SRT changes reflect actual reflex response thresholds rather than random sampling variability. While this method assumes independence between measurements at different time points, it offers a practical means of assessing significant changes within the constraints of our study (Mittal et al., 2019).

## 3 Results

The primary objective was to determine whether the Linmot^®^ tapper could accurately track changes in the stretch reflex threshold following cyproheptadine HCl administration. We conducted four tests: one pre-drug, and three post-drug, measuring reflex force and RIEMG from the biceps brachii using progressive tendon indentation and tapping. Participants, recruited based on inclusion criteria requiring a MAS score of ≥1 (as detailed in Section 2), exhibited MAS scores ranging from 1+ to 3. This variability reflects the inherent heterogeneity of spasticity severity in chronic stroke survivors. Additionally, the MAS grade was also recorded by a physical therapist before each Linmot^®^ tapper test.

**Figure 3:**
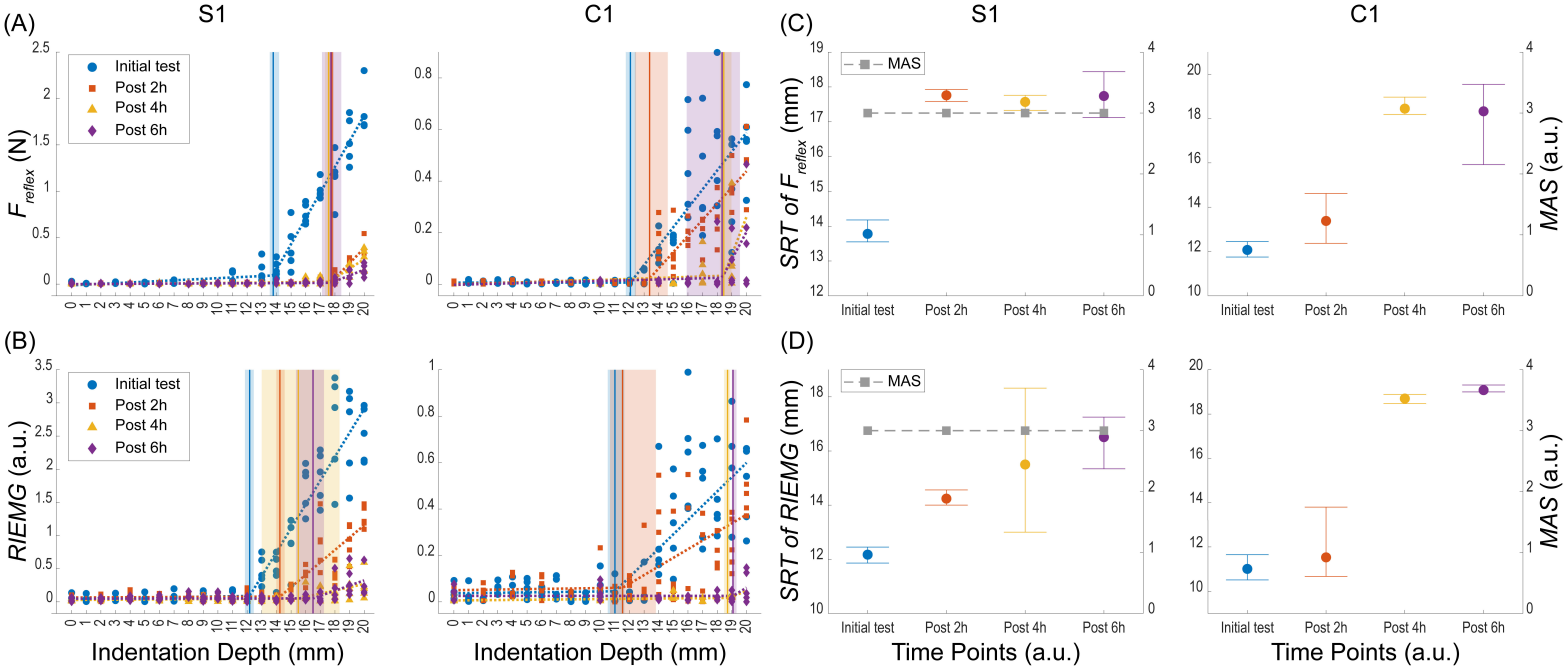
Reflex force and RIEMG results from stroke survivor (S1) and control (C1) subject. **(A)** Reflex Force (*F*_reflex_) results across different indentation depths at various time points. **(B)** RIEMG results across different indentation depths at various time points. Both results were measured at four time points: Initial test (blue circles), Post 2 h (orange squares), Post 4 h (yellow triangles), and Post 6 h (purple diamonds). The dashed lines represent the piecewise linear regression fit using a permutation-based bootstrapping method. The shaded areas represent the width of the 95 percent confidence intervals (CIs) for the estimated change-points (thresholds) indicated as vertical lines. **(C)** Changes in Stretch Reflex Threshold (SRT) of *F*_reflex_ and Modified Ashworth Scale (MAS) grade at four time points. SRT values (left *y*-axis) are represented by colored markers, with error bars indicating the 95% CIs (lower and upper bounds). MAS grades (right *y*-axis) are represented by gray squares connected with dashed lines. **(D)** SRT of RIEMG and MAS grade at four time points. Reflex force (*F*_reflex_) is measured in Newtons (N), and RIEMG is presented in arbitrary units (a.u.). Indentation depths and SRT are presented in millimeters (mm).

Using a bootstrapping technique, we estimated the SRT value for each time point (Figure 3). The mean change points and 95% confidence intervals were derived from 100,000 iterations, and non-overlapping intervals indicated significant differences between time points. Table 1 summarizes the piecewise regression results for the reflex force and RIEMG thresholds before and after medication for four subjects (S1, S2, S3, and C1) with MAS grade.

**Table 1:** Piecewise regression results: threshold estimates of reflex force and RIEMG.

**Subject**	**Time points**	**Threshold (mm) of reflex force**			**Threshold (mm) of RIEMG**			**MAS for biceps**
		**Estimate (SD)**	**95% Confidence interval**		**Estimate (SD)**	**95% Confidence interval**		
			**Lower**	**Upper**		**Lower**	**Upper**	
S1	Initial test	13.78 (0.14)	13.55	14.18	12.17 (0.19)	11.86	12.45	3
	Post-2 h	17.76 (0.10)	17.58	17.93	14.24 (0.16)	14	14.56	3
	Post-4 h	17.57 (0.12)	17.33	17.76	15.50 (1.47)	13	18.32	3
	Post-6 h	17.74 (0.39)	17.12	18.44	16.51 (0.46)	15.34	17.25	3
S2	Initial test	5.52 (0.88)	4.43	7.16	3.78 (3.36)	1.98	12	1+
	Post-2 h	5.78 (0.17)	5.44	6.13	4.13 (1.31)	3.29	4.7	1+
	Post-4 h	10.66 (0.35)	10.22	11.36	6.71 (2.51)	4.3	11	1+
	Post-6 h	NA	NA	NA	NA	NA	NA	NA
S3	Initial test	11.37 (0.36)	10.94	12.7	12.57 (0.29)	12	13.14	2–3
	Post-2 h	14.97 (1.63)	12.89	18.9	15.76 (1.59)	13.33	17.7	2
	Post-4 h	16.39 (0.46)	15.98	17.51	17.93 (0.35)	17	18.54	2–3
	Post-6 h	20.04 (0.01)	20.02	20.06	19.90 (0.07)	19.75	19.99	2
C1	Initial test	12.07 (0.18)	11.75	12.45	11.01 (0.28)	10.51	11.65	NA
	Post-2 h	13.38 (0.80)	12.37	14.62	11.53 (1.20)	10.67	13.8	NA
	Post-4 h	18.45 (0.24)	18.19	18.97	18.70 (0.10)	18.47	18.88	NA
	Post-6 h	18.33 (0.84)	15.92	19.55	19.08 (0.10)	19	19.31	NA

S, Stroke; C, Control. Estimates are expressed as mean (standard error). Threshold values are in millimeters (mm). NA indicates data not applicable; for S2 at Post-6 h, the participant discontinued testing due to scheduling constraints. Threshold estimates were obtained using a permutation-based bootstrapping technique with 100,000 iterations. The mean and standard deviation of the bootstrapped threshold estimates are reported, with 95% confidence intervals calculated from the distribution of the bootstrapped estimates.

### 3.1 Reflex force

S1 showed significant increases in reflex force threshold estimates at all post-medication time points compared to the pre-medication baseline. Specifically, the initial test had a threshold of 13.78 mm (95% CI: 13.55, 14.18). At post-2 h, the threshold significantly increased to 17.76 mm (95% CI: 17.58, 17.93) and remained elevated at post-4 h (17.57 mm, 95% CI: 17.33, 17.76) and post-6 h (17.74 mm, 95% CI: 17.12, 18.44), indicating a sustained effect of the medication. Throughout all tests, the MAS grade remained constant at 3, showing no change in the level of spasticity.

S2 showed no significant change in threshold estimates at post-2 h compared to the initial test, as indicated by overlapping CI (initial threshold: 5.52 mm; 95% CI: 4.43, 7.16; post-2 h threshold: 5.78 mm; 95% CI: 5.44, 6.13). However, at post-4 h, the threshold increased significantly to 10.66 mm (95% CI: 10.22, 11.36) compared to both the initial test and post-2 h. S2 discontinued testing at post-6 h due to the subject's scheduling constraints, so data was not collected at post 6-h. Throughout the tests, the MAS grade for S2 remained consistently at 1+.

S3 exhibited significant increases in the threshold at all post-medication time points compared to the pre-medication baseline. The initial threshold was 11.37 mm (95% CI: 10.94, 12.70). At post-2 h, the threshold significantly increased to 14.97 mm (95% CI: 12.89, 18.90), and this increase continued at post-4 h (16.39 mm, 95% CI: 15.98, 17.51). By post-6 h, the threshold increased to 20.04 mm (95% CI: 20.02, 20.06). The MAS grade fluctuated: from 2 to 3 during the initial test, dropping to 2 at post-2 h, rising to 2 to 3 at post-4 h, and returning to 2 at post-6 h.

C1 showed no significant change in the threshold at post-2 h compared to the initial test, as indicated by overlapping CIs (initial threshold: 12.07 mm; 95% CI: 11.75, 12.45; post-2 h threshold: 13.38 mm; 95% CI: 12.37, 14.62). However, at post-4 h, the threshold increased significantly to 18.45 mm (95% CI: 18.19, 18.97) and remained elevated at post-6 h (18.33 mm, 95% CI: 15.92, 19.55).

### 3.2 RIEMG

S1 showed significant increases in RIEMG threshold estimates at all post-medication time points compared to the pre-medication baseline (Table 1). The initial threshold was 12.17 mm (95% CI: 11.86, 12.45). At post-2 h, the threshold significantly increased to 14.24 mm (95% CI: 14.00, 14.56) and continued to rise at post-4 h to 15.50 mm (95% CI: 13.00, 18.32). By post-6 h, the threshold increased to 16.51 mm (95% CI: 15.34, 17.25).

S2 showed no significant change in thresholds at post-2 h and post-4 h compared to the initial test, as indicated by overlapping CIs. The initial threshold was 3.78 mm (95% CI: 1.98, 12.00). At post-2 h, the threshold slightly increased to 4.13 mm (95% CI: 3.29, 4.70), but the confidence intervals overlapped. Similarly, at post-4 h, the threshold increased to 6.71 mm (95% CI: 4.30, 11.00) with overlapping CIs. Data for post-6 h were unavailable as S2 discontinued participation.

S3 exhibited significant threshold increases at all post-medication time points compared to the initial baseline. The initial threshold was 12.57 mm (95% CI: 12.00, 13.14). At post-2 h, the threshold significantly increased to 15.76 mm (95% CI: 13.33, 17.70). This increase continued at post-4 h (17.93 mm; 95% CI: 17.00, 18.54) and further at post-6 h (19.90 mm; 95% CI: 19.75, 19.99).

C1 showed no significant change in thresholds at post-2 h compared to the initial test, as indicated by overlapping CIs. The initial threshold was 11.01 mm (95% CI: 10.51, 11.65). At post-2 h, the threshold slightly increased to 11.53 mm (95% CI: 10.67, 13.80), but the CIs overlapped. However, by post-4 h, the threshold increased significantly to 18.70 mm (95% CI: 18.47, 18.88), and this increase persisted at post-6 h (19.08 mm, 95% CI: 19.00, 19.31).

### 3.3 Correlation between reflex force and RIEMG thresholds

A high correlation of 0.95 between reflex force and RIEMG thresholds suggests a strong alignment between these two measures. Reflex force thresholds were, on average, 0.95 mm lower than RIEMG thresholds (SD = 1.59 mm), indicating greater sensitivity in recording reflex responses with force measurements. RIEMG estimates showed more variability, with a broader range of confidence intervals (mean difference of 0.99 mm, SD = 2.7 mm), suggesting that RIEMG captures more variability than force estimates.

## 4 Discussion

This study assessed whether the Linmot^®^ tapper can reliably track changes in stretch reflex threshold following drug administration in chronic stroke survivors with spasticity. Overall, the trends show that the reflex threshold, as measured by force and EMG, significantly increased with respect to the onset of drug absorption at each measurement times (2, 4, and 6 h post drug). Our results also revealed a high correlation (0.95) between reflex thresholds as measured by force and RIEMG, with reflex force measures proving more sensitive in detecting spasticity changes. Finally, and most importantly, the Linmot^®^ tapper captured these subtle changes precisely. At the same time, the MAS, a qualitative clinical assessment, was limited to detecting these variations (Table 1), emphasizing the sensitivity and accuracy of the Linmot^®^ tapper for assessing spasticity dynamics.

The analysis revealed that both reflex force and RIEMG thresholds showed significant increases in all subjects following drug administration, indicating the effectiveness of cyproheptadine HCl in modulating spasticity. The time sections that showed significant changes in threshold were consistent across both measures, with a mean threshold value difference within 1 mm and a standard deviation of 1.6 mm. Additionally, the mean range of confidence interval differences was within 1 mm, with a standard deviation of 2.7 mm, suggesting high similarity between threshold values from both measures. This finding aligns with previous studies that reported cyproheptadine HCl's effectiveness on spasticity-like phenomena such as clonus and spasm (Wainberg et al., 1990) and its impact on involuntary hypertonicity by significantly reducing the relaxation time of finger flexor muscles (Kamper et al., 2022). However, these studies did not directly measure spasticity reduction. However, our study is unique in that it tracked the changes in spasticity over time following drug administration. As an antagonist to serotonin by blocking its receptors, cyproheptadine HCl is believed to reduce motor neuron excitability, potentially decrease excessive muscle activity, and help manage symptoms of spasticity (Seo et al., 2011).

Although all subjects underwent the same single dose of cyproheptadine HCl, the drug's effects varied among individuals, indicating subject-dependent interactions and metabolism. Specifically, S1 and S3 showed significant threshold increases between the initial test and post-2 h, while S2 and C1 showed significant increases between post-2 h and post-4 h. For example, reflex force measures showed threshold differences of 3.98, 4.88, 3.6, and 5.07 mm, and RIEMG measures showed differences of 2.07, 2.58, 3.19, and 7.17 mm for S1, S2, S3, and C1, respectively. Moreover, significant changes persisted or increased over time, with S3 showing an additional notable increase (3.65 mm from reflex force and 1.97 mm from RIEMG) after the initial change. Notably, the threshold standard deviations in stroke and control were similar (0.01–1.63 mm for reflex force and 0.07–1.59 mm for RIEMG), indicating minimal baseline variability. C1's threshold changes following cyproheptadine administration further suggest that the drug's effects on the stretch reflex are not solely dependent on the presence of spasticity. Overall, the individual patterns of threshold changes indicate that we can assess when the medication's effects begin to appear, how long they last, and their impact on each stroke survivor. These findings underscore the need for personalized approaches in spasticity management, as individual variability in drug response, metabolism, and treatment duration can significantly impact therapeutic outcomes.

Our results also suggest that threshold estimation from force measures might be as good as or even better than EMG measures for monitoring changes in spasticity. First, the reflex threshold results were similar when using either force or EMG, with force measures detecting significant threshold changes across all participants. The force measure can also provide more information, such as passive mechanical properties of the biceps tendon and surrounding tissues. This data is valuable for evaluating the mechanical characteristics of a tendon and arm, offering insights beyond what EMG can provide.

Force measurement offers practical advantages, requiring no skin preparation or electrode costs, making it convenient and cost-effective. Additionally, force measurements are based on an absolute scale, and therefore, repeat measures will demonstrate less variability and more reliability than RIEMG estimates, suggesting a more consistent and reliable method. The mean difference of −0.95 mm, with a standard deviation of 1.59 mm between the two methods, indicates that reflex force thresholds tend to be slightly lower than RIEMG thresholds. This lower variability supports the notion that reflex force provides a more stable assessment. Considering clinical applications, reflex force measurements are more practical as they eliminate the need to attach and prepare EMG sensors. This convenience, combined with consistent and reliable results, makes reflex force measurement a superior choice for tracking changes in spasticity. In summary, while force and EMG measures are valuable, our findings demonstrate that force measurement offers greater reliability, practicality, and deeper mechanical insights for spasticity assessment.

The scope of this study was limited to characterize short-term variations in stretch reflex threshold (SRT) following cyproheptadine HCI. While these changes provide valuable insights into the drug's mechanism of action and its effects on the stretch reflex, they do not directly assess the impact on limb function or quality of life. Assessment of spasticity with high resolution can improve the precision of a pharmacological intervention on an individual basis. This could supplement standard clinical spasticity assessments, which may not provide the needed resolution. Future research should investigate the relationship between SRT changes and clinical measures of motor function and patient quality of life. Additionally, while the Linmot^®^ tapper provides a more precise alternative to conventional clinical assessments (e.g., MAS), its current complexity may limit practical clinical use. Because the device requires a complex setup with precise alignment, it is impractical for routine clinical application. We are currently working on making the device more portable, simplifying its use, and standardizing protocols to enhance clinical feasibility by adding a mini ultrasound probe at the tapper end for ease of positioning and online feedback that will reduce the measurement times.

## 5 Conclusions

The Linmot^®^ tapper, a tool that measures the muscle stretch reflex threshold by systematically assessing the deep tendon reflex, provides a promising and highly sensitive measure of spasticity. In this study, we tested the potential of Linmot^®^ tapper as a valuable tool for monitoring spasticity after drug administration in clinical settings. Our findings demonstrate that Linmot^®^ tapper can effectively track changes in stretch reflex threshold over time following cyproheptadine HCl administration. Although individual differences were observed, the reflex thresholds estimated by both reflex force and RIEMG showed significant changes 2, 4, and 6 h after drug administration, suggesting that this technique can track the effect of cyproheptadine HCl over time. These results also contribute to our understanding of monoaminergic contributions to the stretch reflex response threshold in hemiparetic stroke survivors and highlight the potential clinical applications of Linmot^®^ tapper in spasticity assessment. Reducing serotonin levels following cyproheptadine HCl administration may reduce spinal motor neuron excitability, decreasing excessive muscle activity and increasing the threshold over time. Comparative systemic studies with conventional methods, such as the MAS, are necessary to evaluate the resolution, reliability, and ability of Linmot^®^ tapper to track spasticity levels after drug administration. Additionally, further research is needed to explore the clinical implications of Linmot^®^ tapper, including making it portable and developing a streamlined assessment protocol.

## Data Availability

The raw data supporting the conclusions of this article will be made available by the authors, without undue reservation.

## References

[B1] Alibiglou L. Rymer W. Z. Harvey R. L. Mirbagheri M. M. (2008). The relation between Ashworth scores and neuromechanical measurements of spasticity following stroke. J. Neuroeng. Rehabil. 5:18. 10.1186/1743-0003-5-18 18627628 PMC2515334

[B2] Ashworth B. (1964). Preliminary trial of carisoprodol in multiple sclerosis. Practitioner 192, 540–542. 14143329

[B3] Calota A. Feldman A. G. Levin M. F. (2008). Spasticity measurement based on tonic stretch reflex threshold in stroke using a portable device. Clin. Neurophysiol. 119, 2329–2337. 10.1016/j.clinph.2008.07.215 18762451

[B4] Centen A. Lowrey C. R. Scott S. H. Yeh T. T. Mochizuki G. (2017). KAPS (kinematic assessment of passive stretch): a tool to assess elbow flexor and extensor spasticity after stroke using a robotic exoskeleton. J. Neuroeng. Rehabil. 14:59. 10.1186/s12984-017-0272-8 28629415 PMC5477344

[B5] Chardon M. K. Dhaher Y. Y. Suresh N. I. Jaramillo G. Rymer W. Z. (2015). Estimation of musculotendon kinematics under controlled tendon indentation. J. Biomech. 48, 3568–3576. 10.1016/j.jbiomech.2015.07.024 26321363 PMC4816076

[B6] Chardon M. K. Rymer W. Z. Suresh N. L. (2014). Quantifying the deep tendon reflex using varying tendon indentation depths: applications to spasticity. IEEE Trans. Neural. Syst. Rehabil. Eng. 22, 280–289. 10.1109/TNSRE.2014.2299753 24621852

[B7] Chardon M. K. Suresh N. L. Dhaher Y. Y. Rymer W. Z. (2020). *In-vivo* study of passive musculotendon mechanics in chronic hemispheric stroke survivors. IEEE Trans. Neural Syst. Rehabil. Eng. 28, 1022–1031. 10.1109/TNSRE.2020.2972206 32149642 PMC7233468

[B8] Choi S. Shin Y. B. Kim S. Y. Kim J. (2018). A novel sensor-based assessment of lower limb spasticity in children with cerebral palsy. J. Neuroeng. Rehabil. 15:45. 10.1186/s12984-018-0388-5 29866177 PMC5987429

[B9] Condliffe E. G. Clark D. J. Patten C. (2005). Reliability of elbow stretch reflex assessment in chronic post-stroke hemiparesis. Clin. Neurophysiol. 116, 1870–1878. 10.1016/j.clinph.2005.02.030 15979400

[B10] Dehem S. Gilliaux M. Lejeune T. Detrembleur C. Galinski D. Sapin J.. (2017). Assessment of upper limb spasticity in stroke patients using the robotic device REAplan. J. Rehabil. Med. 49, 565–571. 10.2340/16501977-2248 28664214

[B11] Feldman A. G. (2015). Referent Control of Action and Perception. Challenging Conventional Theories in Behavioral Neuroscience. New York, NY: Springer. 10.1007/978-1-4939-2736-4

[B12] Haugh A. B. Pandyan A. D. Johnson G. R. (2006). A systematic review of the Tardieu scale for the measurement of spasticity. Disabil. Rehabil. 28, 899–907. 10.1080/09638280500404305 16861197

[B13] Hornby T. G. McDonagh J. C. Reinking R. M. Stuart D. G. (2002). Effects of excitatory modulation on intrinsic properties of turtle motoneurons. J. Neurophysiol. 88, 86–97. 10.1152/jn.2002.88.1.86 12091534

[B14] Kamper D. Barry A. Bansal N. Stoykov M. E. Triandafilou K. Vidakovic L.. (2022). Use of cyproheptadine hydrochloride (HCl) to reduce neuromuscular hypertonicity in stroke survivors: a randomized trial: reducing hypertonicity in stroke. J. Stroke Cerebrovasc. Dis. 31:106724. 10.1016/j.jstrokecerebrovasdis.2022.106724 36054974 PMC9533231

[B15] Lance J. W. (1980). The control of muscle tone, reflexes, and movement: Robert Wartenbeg Lecture. Neurology 30, 1303–1303. 10.1212/WNL.30.12.1303 7192811

[B16] Lemoyne R. Mastroianni T. Kale H. Luna J. Stewart J. Elliot S.. (2011). Fourth generation wireless reflex quantification system for acquiring tendon reflex response and latency. J. Mech. Med. Biol. 11, 31–54. 10.1142/S0219519410003654

[B17] Li S. Francisco G. E. Rymer W. Z. (2021). A new definition of poststroke spasticity and the interference of spasticity with motor recovery from acute to chronic stages. Neurorehabil. Neural Repair 35, 601–610. 10.1177/15459683211011214 33978513

[B18] Mittal N. Bhandari M. Kumbhare D. (2019). A tale of confusion from overlapping confidence intervals. Am. J. Phys. Med. Rehabil. 98, 81–83. 10.1097/PHM.0000000000001016 30119088

[B19] Mooney C. Z. Duval R. D. Duvall R. (1993). Bootstrapping: A Nonparametric Approach to Statistical Inference. Newbury Park, CA: Sage.

[B20] Nance P. W. (1994). A comparison of clonidine, cyproheptadine and baclofen in spastic spinal cord injured patients. J. Am. Paraplegia Soc. 17, 150–156. 10.1080/01952307.1994.11735927 7964712

[B21] Pandyan A. Gregoric M. Barnes M. Wood D. Wijck F. v. Burridge J. Hermens H. Johnson G. (2005). Spasticity: clinical perceptions, neurological realities and meaningful measurement. Disabil. Rehabil. 27, 2–6. 10.1080/09638280400014576 15799140

[B22] Park J. H. Kim Y. Lee K. J. Yoon Y. S. Kang S. H. Kim H.. (2019). Artificial neural network learns clinical assessment of spasticity in modified Ashworth scale. Arch. Phys. Med. Rehabil. 100, 1907–1915. 10.1016/j.apmr.2019.03.016 31009599

[B23] Raptis H. Burtet L. Forget R. Feldman A. G. (2010). Control of wrist position and muscle relaxation by shifting spatial frames of reference for motoneuronal recruitment: possible involvement of corticospinal pathways. J. Physiol. 588, 1551–1570. 10.1113/jphysiol.2009.186858 20231141 PMC2876809

[B24] Seo N. J. Fischer H. W. Bogey R. A. Rymer W. Z. Kamper D. G. (2011). Effect of a serotonin antagonist on delay in grip muscle relaxation for persons with chronic hemiparetic stroke. Clin. Neurophysiol. 122, 796–802. 10.1016/j.clinph.2010.10.035 21075681

[B25] Sin M. Kim W. S. Cho K. Paik N. J. (2019). Isokinetic robotic device to improve test-retest and inter-rater reliability for stretch reflex measurements in stroke patients with spasticity. J. Vis. Exp. 48, e59814. 10.3791/59814-v 31259910

[B26] Starsky A. J. Sangani S. G. McGuire J. R. Logan B. Schmit B. D. (2005). Reliability of biomechanical spasticity measurements at the elbow of people poststroke. Arch. Phys. Med. Rehabil. 86, 1648–1654. 10.1016/j.apmr.2005.03.015 16084821

[B27] Wainberg M. Barbeau H. Gauthier S. (1990). The effects of cyproheptadine on locomotion and on spasticity in patients with spinal cord injuries. J. Neurol. Neurosurg. Psychiatry 53, 754–763. 10.1136/jnnp.53.9.754 2246657 PMC1014252

[B28] Wang M. Y. Dun N. J. (1990). 5-Hydroxytryptamine responses in neonate rat motoneurones *in vitro*. J. Physiol. 430, 87–103. 10.1113/jphysiol.1990.sp018283 2150862 PMC1181729

[B29] Young K. D. Lewis R. J. (1997). What is confidence? Part 1: the use and interpretation of confidence intervals. Ann. Emerg. Med. 30, 307–310. 10.1016/S0196-0644(97)70166-5 9287892

